# The Diseases of the Fœtus

**Published:** 1841-01

**Authors:** 


### Art. XV.

*Die Krankheiten des Foetus.* Von Dr. J. Graetzer.—*Breslau,* 1837. 8vo, pp. 286.
*The Diseases of the Fœtus.* By Dr. J. Graetzer.—*Breslau,* 1837.

In the year 1702 the first treatise on diseases of the fœtus was published under the auspices of the celebrated Hoffmann ; and there have appeared since that time many contributors to this department of medical science. Few, however, have done more than record one or two isolated observations in some journal or other, where they were hidden from the notice of subsequent students rather than made available for the furtherance of their enquiries. The collection of these observations and their suitable arrangement was the laborious task that M. Graetzer undertook, and which he has executed with much diligence and sound judgment. He makes no pretensions, indeed, to have fulfilled every object that might have appeared desirable; nor does he profess to have furnished a complete systematic treatise on intra-uterine pathology ; any attempt at which, in the present state of our knowledge, must have been fruitless. It is but right, however, that we should allow M. Graetzer to state for himself what has been his aim in this work, and by what principles he has been guided in its execution.

"The object of a scientific pathology of the fœtus," says M. Graetzer, "is not merely to state with accuracy the diseases to which the fœtus is liable, but also to show how each disease is modified by the different stages of development of the embryo. Whenever this is accomplished we shall have obtained trustworthy data from which to deduce the real causes of these affections. In spite of the labour of nearly two centuries, however, the execution remains far behind the idea, and makes even now but small advances towards its realization. Many diseases indeed have been observed in the fœtus, and the greater number of those persons who have investigated the subject have not confined themselves to a bare description of what they saw, but have almost invariably attempted to attach to the morbid conditions names such as are applied to diseases of the adult. This, however, has been done apparently without the slightest suspicion of the important pathological questions which are here presented for solution. It would, then, be unwise to attempt now to arrange our materials according to this idea, for our observations do not afford premises from which we might deduce definite results. Our present object, therefore, has been to furnish a complete critical history of all the diseases of the fœtus: a work which has not hitherto been satisfactorily executed." (pp. 4, 5.)

In the arrangement adopted by M. Graetzer two grand classes of general and local diseases are established. The following extract from his scheme will best exhibit the numerous topics treated of in the work, as well as the order in which they are discussed.

"I. General diseases.
*a.* Of an acute character.
1. Fever.

*α.* Unattended with any eruption on the surface of the body: as intermittent fever.

*β.* With an eruption: as the acute exanthemata.

2. Inflammations.
*b.* Chronic diseases.

1. Dyscrasiæ affecting especially internal organs: as hypertrophy, atrophy, syphilis, dropsy, &c.

2. Dyscrasiæ especially affecting the skin: as elephantiasis, and some other cutaneous affections.

II. Local diseases.
*a.* Of the organic fluids.
1. Of the blood: scurvy.
2. Of the lymph: scrofula.
*b.* Organs of vegetative life.
1. Intestinal canal.
2. Its glandular appendages," &c. (pp. 7-8.)

Some of these diseases might perhaps have been better classed under other heads, but M. Graetzer proposes this arrangement merely as a temporary one until increased knowledge may enable us to diminish the class of general diseases, and to refer each affection to the organ whence it derives its origin.

After giving a good sketch of the literature of his subject, the author begins the consideration of the general diseases of the fœtus, with some observations on intermittent fever. (§ 8.) This disease, how problematical soever its occurrence during intra-uterine life may appear, has certainly been observed a very short time after birth. In addition to the cases related by M. Graetzer, we may refer to two instances of congenital intermittent fever recorded in Von Siebold's Journal, (bd. xvii. p. 318.)

Much research is displayed by the author (§§ 9-15) in his remarks upon the smallpox, measles, pemphigus, and petechiæ, in the fœtus; all of which he classes, though with some hesitation, among the exanthemata. The following conclusions may be drawn from the author's observations with reference to variola in the fœtus. 1st, That smallpox may be communicated by the mother to the fœtus. 2d, That the mother may transmit the smallpox to the fœtus without herself suffering from the disease. 3d, That there is no well authenticated instance in which the mother has suffered smallpox and the child has escaped. 4th, That where both mother and child have been attacked by the disease the latter has not been affected until a considerable time after the former. Thus, in the case which Mr. Lynn relates, a pregnant female was attacked by smallpox: on the eleventh day of the disease the pustules dried up; and on the twenty-second day she was delivered of a child. The whole body of the child was covered with the eruption of smallpox, and three days after birth the pustules were distended with matter. From this it would appear that the first symptoms in the child could not have occurred sooner than the sixth day after the pustules in the mother had dried up. The case related by Mr. Hunter, and others observed by different persons, bear out the same conclusion.

Under the head Inflammations (§§ 17-19), the author details cases of spontaneous amputation of the limbs in the fœtus, with the various explanations of the accident which have been proposed.

Among the Dyscrasiæ principally affecting internal organs, hypertrophy, atrophy, syphilis, helminthiasis, lithiasis, hydrops, and icterus are noticed in succession. (§§ 20-30.) By hypertrophy the author understands preternatural development of fat, and might surely have found some more appropriate word to express his meaning. The observations on atrophy are short and unsatisfactory; but M. Graetzer has treated of syphilis at much length (pp. 85-107), and has presented us with a better summary of all facts relating to this interesting subject than we have ever met with elsewhere.

The existence of stone in the kidney or bladder of the fœtus is undoubtedly an exceedingly rare occurrence, though several instances of it have been collected by the diligence of M. Graetzer. (§ 27.) The first case on record, which he quotes from Hoffmann, was that of a daughter of the Princess Moritz of Zeis. The princess, while suffering from stone in the kidney, gave birth to a daughter, who very soon began to suffer from difficulty in voiding her urine. When only three weeks old she died; and, on a post-mortem examination, a stone the size of a peach was found in her bladder. Geyer, Löscke, Nicolai, Feiler, and Prael have likewise related somewhat similar cases. Prael found many calculi in both kidneys of a female child who died when six months old. Most of them were hard, as large as a grain of millet, and contained phosphate of lime, uric acid, and albumen. The child when born was stout and healthy, but suffered from birth from obstinate constipation, and made water but very seldom. Her urine was dark-coloured, and had a very strong urinous smell. Diarrhœa afterwards supervened, and the child died in convulsions. Orfila has observed two instances of stone in the fœtal bladder; and in both of these cases calculi were also present in the pelvis of the kidneys. The bladder was evidently inflamed, and the tissue of the kidneys seemed also to participate in the same condition, if one might judge from its sanguineous congestion, its colour, and extreme friability.

The jaundice of new-born infants is an affection familiar to all who are conversant with the diseases of children; but English writers, with the exception of Underwood, who alludes to it incredulously, appear to be unacquainted with its occurrence in utero. M. Graetzer has applied his usual diligence in the illustration of this subject (§§ 29-30), and has likewise detailed fully M. Lobstein's observations on the disease which he calls Kirrhonosis, and which he distinguishes from ordinary icterus.

M. Lobstein understands by the name Kirrhonosis a disease in which there exists a deep yellow colour of the serous membranes and of the substance of the nerves. It resembles an internal jaundice of the peritoneum, pleura, pericardium, and arachnoid; and differs from ordinary icterus in the circumstance that the subcutaneous cellular tissue and that which penetrates into the parenchyma of the organs, as well as the substance of the organs themselves, are quite free from this yellow colour. These observations, which M. Lobstein first made on two five-months-old fœtuses, he repeated on others, and found that the substance of the nerves was tinged; and that neither washing nor maceration in water or spirit could remove the yellow colour of the serous membranes. We are ignorant of the causes of this affection: it appears to differ from the ordinary icterus of new-born infants only in its situation; but, as M. Andral remarks with truth, it is by no means certain that even there the yellow colour is produced by bile.

The second part of the book, which treats of local affections, opens with a consideration of scurvy and scrofula: the former being regarded as a disease of the blood, the latter of the lymph. (§§ 34-5.) The interesting subject of scrofula and tubercle is, however, treated in a far too cursory manner. Aphtha, inflammation of the œsophagus, stomach, and peritoneum, and umbilical hernia, follow among the diseases of the organs of vegetative life. (§§ 36-42.) The observations on peritonitis are few, and add nothing to what has been so well done by Dr. Simpson in his valuable papers in the Edinburgh Journal. Diseases of the generative organs occupy but a short space. (§§ 46-9.) The author notices congenital inguinal hernia in the male; but the occurrence of hernia of the fallopian tube and ovary in the female seems to have escaped his observation. Notices of this variety of hernia may be found in Deneux, (Recherches sur la Hernie de l'Ovaire, Paris, 1813;) Meckel, (Handbuch der pathologischen Anatomie, bd. ii. p. 429;) Billard, (Traité des Maladies des Enfans Observat. 57, p. 474, and Atlas, Pl. x.;) and Busch, (Neue Zeitschrift für Geburtskunde, bd. viii. heft 2, p. 272.)

Diseases of the circulatory and respiratory systems come next under review. (§§ 50-4.) It is here curious to observe how frequently the lungs which during fœtal life have no distinct function to perform are the seat of disease. These diseases too are various in kind and differ in the part of the organ which they affect. Inflammation of the pleura, terminating in sero-purulent effusion or in the formation of adhesions, is by no means an unusual occurrence. Cruveilhier has noticed the frequency of inflammation of the lungs in the fœtus, and the slight influence upon its nutrition which the disease appears to exert. Tubercles in the lungs are not infrequent, but it is not usual for them to have passed the crude state during intra-uterine life: instances to the contrary, however, are related by Husson and Cruveilhier; and in one instance Lobstein found the lung to contain calcareous concretions. M. Graetzer does not notice tubercular degeneration of the bronchial glands, although it is more frequently met with both in the fœtus and the infant than tubercle in the lung.

In investigating the diseases of the locomotive organs in the fœtus, M. Graetzer treats first of rhachitis (§§ 55-6), with his usual erudition. We pass over his observations on this subject, however, in order to notice his remarks on fracture of the bones occurring before birth (§§ 58-60), an accident of considerable importance in a medico-legal point of view. Eighteen cases of the accident are detailed by M. Graetzer, of which the following, quoted by him from Pr. Carus, is one of the most remarkable:

"A healthy woman, aged twenty-five, fell from a ladder upon her abdomen when in the sixth month of her pregnancy. At the moment of the accident the child's movements became more violent than ordinary; but afterwards they grew weaker, and at the end of her pregnancy she was delivered, after a natural labour completed without manual interference, of a puny and emaciated child, which gave but feeble signs of life. On the right leg of the child was a wound three fourths of an inch in length, which ran transversely from the outer to the inner ancle, and had divided the skin and muscular substance; and the tibia itself was broken at its lower extremity in such a manner that the epiphysis was detached. The lips of the wound were pale, bloodless, and flaccid. The epiphysis was completely detached from the lower end of the tibia which protruded from the wound, was directed outwards, had lost its periosteum, and presented an unhealthy appearance. Reduction was attempted; but the attempt was not persevered in, because the edges of the wound were affected with sphacelus, and necrosis of the bone was evidently advancing. The disease spread and the child died on the thirteenth day. Carus regards this case as a proof that affections may be borne during intra-uterine life, which become speedily fatal after birth. He looks on it likewise as important in a medico-legal point of view; since, had any manual interference been resorted to in the progress of the labour, the suspicion of injury having been inflicted on the child by awkwardness or carelessness might easily have arisen." (pp. 198-9.)

Other cases are related in which fractures in the fœtus have been found completely healed. D'Outrepont mentions having found in a stillborn child the right thigh misshapen and a hard prominence projecting from the middle of its shaft. This induced him to examine the other limbs, when he found similar prominences on both fore-arms and on the left collar bone, and in those situations there was an evident deposition of callus. It seemed, indeed, that this callus had been recently formed, since the union of the fracture of one of the fore-arms was not quite firm.

Injuries to the fœtal skull in its passage through the pelvis naturally arrange themselves here, and accordingly the author treats of them in the next two sections, (§§ 61-2.) He next gives an abstract of Dupuytren's valuable paper on spontaneous luxation of the femur; and he also establishes satisfactorily that the subject had not been altogether unnoticed before the time of that celebrated surgeon. He even quotes (p. 222) a passage from Hippocrates (De Articul., sect. vi., Nos. 26-9), in which this affection is clearly described though not distinguished from hip-joint disease occurring in infancy.

Cephalæmatoma, to an examination of which M. Graetzer devotes §§ 67-8, is an affection which in England has been but little investigated, having been confounded with the ordinary caput succedaneum, and regarded merely as the result of pressure on the head in its passage through the pelvis. This notion, however, was adopted without a due examination of the subject, for the ordinary tumour of the scalp differs greatly from that effusion of blood between the cranial aponeurosis and the skull, or between the bone and its pericranium, to which the name cephalæmatoma is applied. Moreover, the two affections have been known to coexist and to occupy different regions of the head; and cephalæmatoma has been observed in cases where the head met with no difficulty in its passage through the pelvis. The investigations of Michaelis, Paletta, and others have shown that it is often connected with a morbid state of the cranial bones; but this was asserted by some observers to be merely an occasional and accidental consequence of the extravasation of blood. This opinion was supported by Professor Naegele, and Graetzer is in doubt to which side of the question to incline. Since the publication of M. Graetzer's work, however, the subject has been elucidated by the labours of M. Valleix in France, and of M. Burchard in Germany, from which it appears that the cranial bones are implicated in every instance of cephalæmatoma. (See our notice of these works, Vol. VII., pp. 74-87; See also Vol. I., p. 182.)

Cataract is the only affection of the organs of the senses noticed by M. Graetzer; and a few observations on diseases of the cerebro-spinal system (§§77-80), followed by a general recapitulation, conclude the work.

The above notice of the principal contents of this book may convey some notion of the labour and research which it displays, and which render it, in spite of all defects in its arrangement, indispensable to every one engaged in the study of intra-uterine pathology. We trust that M. Graetzer will follow out his original plan, and furnish us, in a second volume, with a view of the diseases of the placenta and other parts of the ovum.

